# Elevated Levels of G-Quadruplex Formation in Human Stomach and Liver Cancer Tissues

**DOI:** 10.1371/journal.pone.0102711

**Published:** 2014-07-17

**Authors:** Giulia Biffi, David Tannahill, Jodi Miller, William J. Howat, Shankar Balasubramanian

**Affiliations:** 1 Cancer Research UK Cambridge Institute, University of Cambridge, Cambridge, United Kingdom; 2 Department of Chemistry, University of Cambridge, Cambridge, United Kingdom; Imperial College London, United Kingdom

## Abstract

Four-stranded G-quadruplex DNA secondary structures have recently been visualized in the nuclei of human cultured cells. Here, we show that BG4, a G-quadruplex-specific antibody, can be used to stain DNA G-quadruplex structures in patient-derived tissues using immunohistochemistry. We observe a significantly elevated number of G-quadruplex-positive nuclei in human cancers of the liver and stomach as compared to background non-neoplastic tissue. Our results suggest that G-quadruplex formation can be detected and measured in patient-derived material and that elevated G-quadruplex formation may be a characteristic of some cancers.

## Introduction

Non-canonical G-quadruplex DNA secondary structures can be formed from guanine-rich DNA sequences of the type G_3_+N_L1_ G_3_+N_L2_ G_3_+N_L3_ G_3_+(N = any base and L = loop) and such sequence motifs are prevalent throughout the human genome [1], [2]. G-quadruplexes are four-stranded structures that comprise stacks of tetrads formed through Hoogsteen hydrogen-bonding of four guanines coordinated by a central monovalent cation [3]. Oligonucleotides folded into a G-quadruplex structure *in vitro* have a high thermodynamic stability under near-physiological conditions, consistent with their formation in cells [4]. The predicted location of G-quadruplex sequence motifs in regulatory regions of the genome, such as promoters and telomeres, suggests that G-quadruplex structures may have important roles in genome function [5]–[7]. A number of helicases that resolve G-quadruplexes have been identified and are hypothesized to contribute to normal genome functions, such as replication, transcription and the maintenance of genome stability [8]. The visualization of DNA G-quadruplex structures in human cells has supported the existence of G-quadruplexes in the genome [9]. In these experiments, a highly selective G-quadruplex-specific antibody (BG4) was generated by phage display and employed to visualize discrete foci of G-quadruplex structures in the nuclei of human tissue culture cell lines. This antibody has provided an opportunity to probe the cellular role of G-quadruplexes and also their potential involvement in disease.

G-quadruplex structures have been suggested to be associated with cellular functions such as replication and transcription. For example, G-quadruplex stabilization by a small molecule can repress transcription of certain oncogenes and/or induce DNA damage at telomeres and oncogenes leading to replication defects and cell death [10]–[17]. G-quadruplex structures may also be linked to genome instability and G-quadruplex sequence motifs have been found proximal to DNA breakpoints and sites of somatic copy number changes seen in several cancers [18], [19]. Helicases that resolve G-quadruplexes are also found localized at sites of genome instability [20]–[22], and diseases such as Bloom’s and Werner’s syndromes, which show increased levels of genome instability and a predisposition to cancer, have contributing mutations in G-quadruplex-resolving helicases [23].

Given the potential relationship between G-quadruplex structures and human disease, it is an important goal to address whether G-quadruplexes can be detected within human tissues. We therefore investigated the detection of DNA G-quadruplexes in patient-derived tissues and whether there are differences in G-quadruplex levels between non-neoplastic and cancer tissues. By employing the G-quadruplex-specific antibody, BG4, in immunohistochemistry with formalin-fixed paraffin-embedded (FFPE) tissue microarray sections, we have detected widespread DNA G-quadruplex formation in the nuclei of human tissues. Quantification of BG4-positive cell nuclei revealed more extensive formation of G-quadruplex structures in stomach and liver cancers compared to the corresponding background non-neoplastic tissues. These results suggest that processing of G-quadruplex structures is mis-regulated in some human cancers.

## Results and Discussion

**Figure 1:**
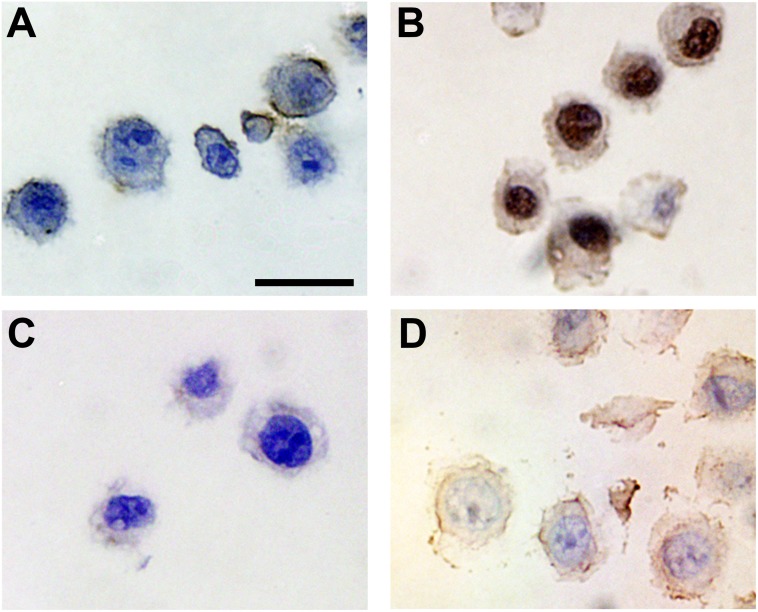
Validation of G-quadruplex nuclear staining by immunohistochemistry. **A**. Human MDA-MB-231 breast adenocarcinoma cell pellets were fixed, paraffin embedded and processed for IHC using the G-quadruplex-specific antibody BG4. No staining is observed in the absence of epitope retrieval. Scale bar corresponds to 20 µm. Nuclei are counterstained with haematoxylin (blue). **B**. Strong BG4 staining (brown) is readily apparent in cell nuclei following antigen retrieval with citrate-based buffer pH 6.0. **C**. No staining is observed in the absence of the BG4 primary antibody. **D**. No staining is seen following DNase treatment prior to BG4 staining. These results show that BG4 can be employed for DNA G-quadruplex detection by IHC staining.

We set out to demonstrate the presence of G-quadruplexes in the nuclei of human tissues and to investigate whether any differences are readily apparent in patient-derived cancer tissues. Our approach was based on the detection of nuclear G-quadruplexes by immunohistochemistry (IHC) with the G-quadruplex-specific antibody, BG4, on non-neoplastic and cancer tissue microarrays (TMAs). The specificity and selectivity of BG4 for G-quadruplex structures and the application of BG4 in immunofluorescence (IF) microscopy on human cells have been previously described [9]. To determine the suitability of BG4 for IHC, we first tested this antibody in a model system using sections from FFPE cell pellets of MDA-MB-231 breast cancer cells that were previously observed to display nuclear G-quadruplex foci by IF microscopy [9]. As epitope retrieval, either heat-mediated (in citrate-based pH 6.0 or Tris/EDTA-based pH 9.0 buffers) or enzymatic (with proteinase K), is often required to expose antigenic sites before antibody binding, we compared these three standard epitope retrieval pre-treatments. After BG4 incubation, staining was performed by incubation with a secondary anti-FLAG antibody, which recognizes a FLAG tag epitope present in the BG4 antibody, followed by Leica horseradish peroxidase polymer-based detection using the Bond IHC staining platform. Following this protocol, intense nuclear staining was observed with BG4 only after epitope retrieval (Figure 1B and S1). No staining was observed in the absence of pre-treatment (Figure 1A) and controls performed in the absence of BG4 showed that epitope retrieval with citrate-based buffer gave less background than that with Tris/EDTA (Figure 1C and S1), whereas pre-treatment with proteinase K led to non-specific nuclear staining even in the absence of BG4 (Figure S1). Moreover, after epitope retrieval with citrate or Tris/EDTA buffers, DNase I treatment prior to BG4 staining led to a strong reduction in BG4 nuclear signal intensity (Figure 1D and S1), further confirming the detection of DNA G-quadruplexes. The nuclear BG4 signal seen in IHC is consistent with the previous detection of BG4 foci in the nuclei of tissue culture cells [9].

**Figure 2:**
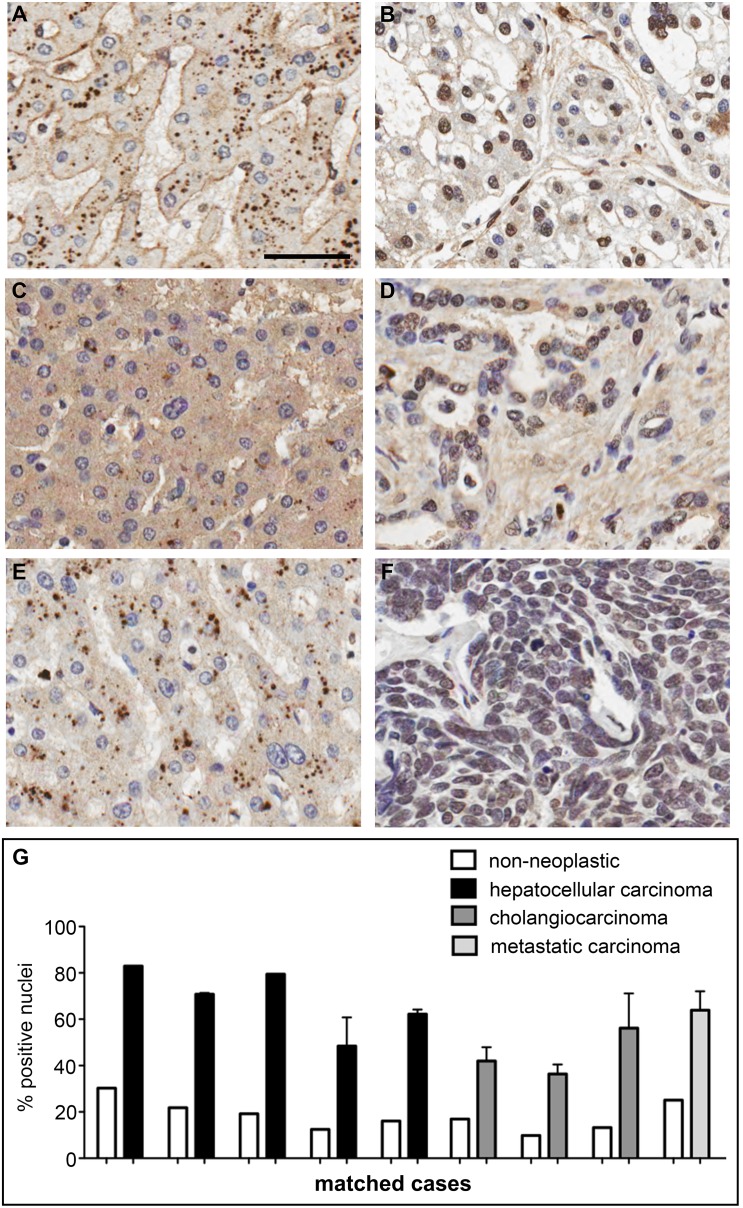
Increased incidence of DNA G-quadruplex structures in human liver cancer relative to matched non-neoplastic tissue. Background non-neoplastic and cancer liver tissues were stained by IHC using the G-quadruplex-specific antibody BG4. **A**. The nuclei of non-neoplastic liver tissue from a hepatocellular carcinoma patient are mostly BG4-negative, with haematoxylin counterstaining (blue) evident. Scale bar corresponds to 50 µm. Note that the cytoplasmic granules are variable and likely to be heme-based artifacts sometimes observed in liver IHC. **B**. Neoplastic tissue from the same patient in **A** shows the extensive presence of BG4-positive nuclei (brown). **C**. The nuclei of non-neoplastic liver tissue for an intrahepatic cholangiocarcinoma patient are mostly BG4-negative. **D**. Neoplastic tissue from the same patient in **C** shows many BG4-positive nuclei. **E**. The nuclei of non-neoplastic liver tissue from a metastatic large cell undifferentiated lung carcinoma patient are mostly BG4-negative. **F**. Metastatic liver tissue isolated from the same patient in **E** shows many BG4-positive nuclei. **G**. Quantification of the number of BG4-positive nuclei in liver cancer and corresponding matched non-neoplastic tissues from nine patients. Error bars represent the standard error of the mean (s.e.m.) calculated when duplicate TMA cores were available. Overall, these results indicate that there are a greater number of G-quadruplex structures in liver cancer compared to background non-neoplastic tissue from the same individual.

**Figure 3:**
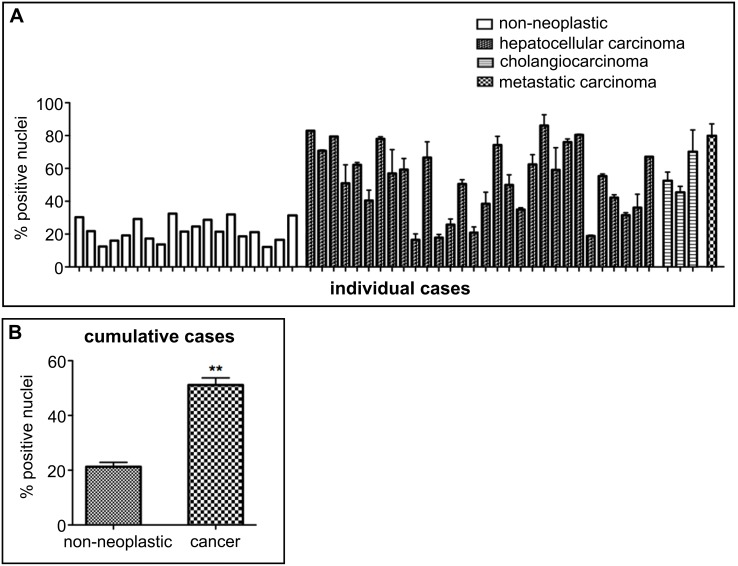
Increased incidence of G-quadruplex-positive cell nuclei across human liver cancers. **A**. Quantification of the number of BG4-positive nuclei in individual liver tissues including unmatched TMA cores. Non-neoplastic liver shows less BG4-positive nuclei compared to hepatocellular carcinoma or intrahepatic cholangiocarcinoma. Each column corresponds to a single tissue sample and error bars represent the s.e.m. calculated when duplicate tissue samples were available. **B**. Overall quantification of the number of BG4-positive nuclei in all non-neoplastic and cancer liver tissues. Error bars represent the s.e.m. **P<0.001, n = 19 and 32 for non-neoplastic and cancer cores, respectively. These results confirm the generality of more extensive G-quadruplex formation in the liver cancers compared to non-neoplastic tissues.

Having established an IHC protocol for BG4, we first confirmed that biopsied non-neoplastic human tissue samples could be stained using BG4. Generally, we observed varied BG4 staining patterns across many tissues suggesting differences in the formation of G-quadruplexes between tissues and also between cells within the same tissue (Figure S2). These results indicate that the overall formation of DNA G-quadruplexes in complex human tissues can be assessed using BG4 in IHC, thereby extending the visualization of G-quadruplex structures beyond IF microscopy in tissue culture cells. In a provisional survey of BG4 staining on cancer tissues compared with background non-neoplastic tissue, differences in BG4 staining intensity or distribution appeared mostly inconclusive (data not shown), and in many cases rigorous quantification was not possible due to significant differences in morphology and presence of multiple cell types in malignant versus non-neoplastic tissue. In contrast, for liver and stomach, our provisional examination suggested that these tissues were readily quantifiable due to a more uniform appearance within and between non-neoplastic and cancer tissues. We therefore used the Aperio Imagescope Nuclear (v9) image analysis software to quantify the percentage of BG4-positive nuclei present in the TMAs. A separate image analysis classifier was developed for each tissue type to accurately identify and separate touching objects, with the malignant and non-neoplastic samples scored using the same parameters for accurate comparison. Strikingly, in nine cases for which we had duplicate liver cancer TMA cores with the corresponding matched background non-neoplastic tissue taken from the same patient, we observed a far greater number of BG4-positive nuclei in cancer (mean 60.3±5.4%) versus non-neoplastic (mean 18.3±2.12%) tissue cores (Figure 2). When individual cancer phenotypes were examined, we found that both hepatocellular carcinoma (HCC) and intrahepatic cholangiocarcinoma (ICC) showed significantly greater nuclear BG4 staining compared to non-neoplastic tissue (Figure 2A–D and G). Although HCC and ICC have different cellular origins, developing from hepatocytes or bile duct cholangiocytes respectively, both showed a greater number and intensity of BG4-positive nuclei compared to the non-neoplastic liver tissue. Furthermore, in metastases (isolated from large cell undifferentiated lung carcinoma sites) an increase in BG4-positive staining was also noted (Figure 2E–G). We observed clear differences between non-neoplastic and tumor tissue for a number of different patient cases. Indeed, a wider-ranging analysis of cancer and background non-neoplastic TMA cores including unmatched cases again showed an increase in BG4-positive nuclei across HCC, ICC and metastatic carcinoma (Figure 3A). When all cases were considered together, a ∼2.4-fold increase in the number of BG4-positive nuclei was seen in liver cancer compared to non-neoplastic tissue (P<0.001, Figure 3B).

**Figure 4:**
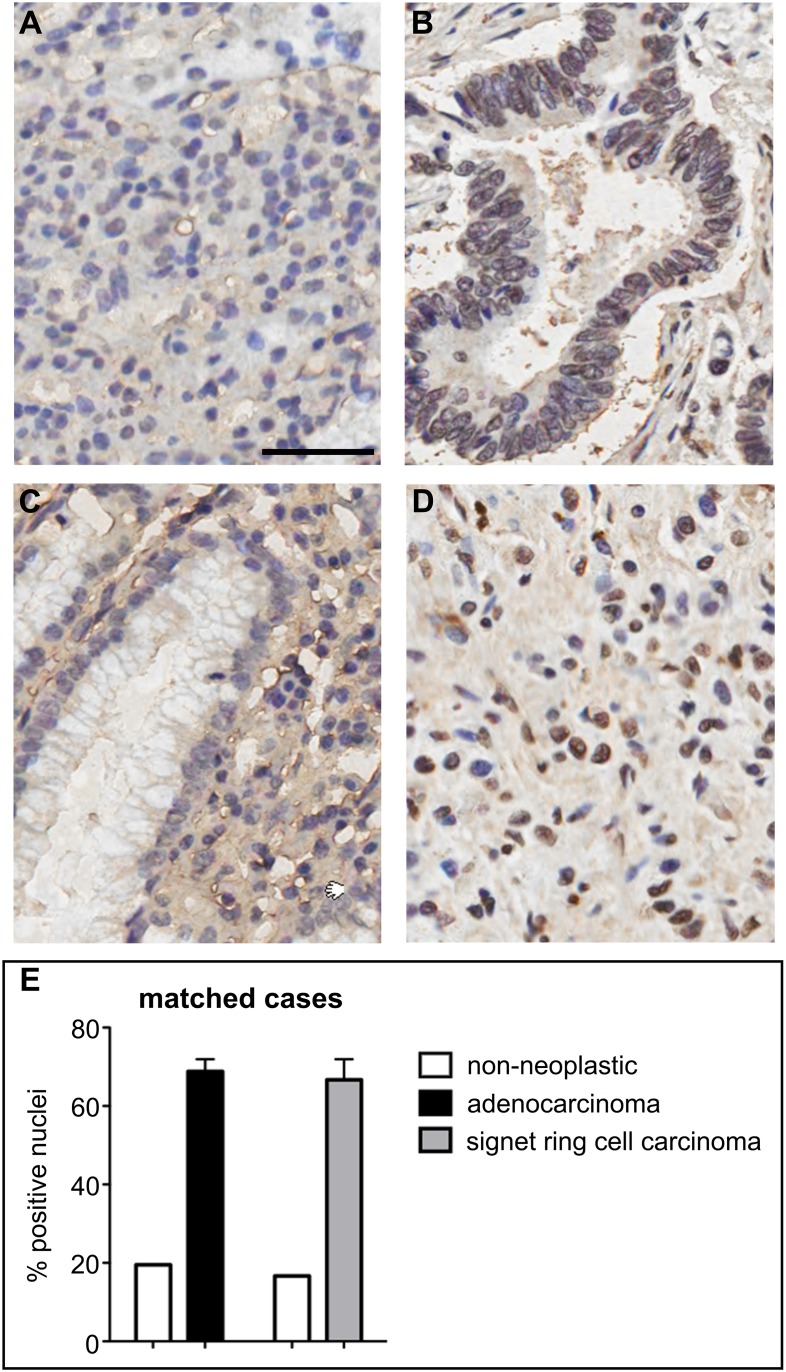
Increased incidence of DNA G-quadruplex structures in human stomach cancer relative to matched non-neoplastic tissue. Gastric adenocarcinoma and signet ring cell carcinoma and matched background non-neoplastic tissues taken from the same patient were stained with the G-quadruplex-specific antibody BG4 by IHC. **A**. The nuclei of non-neoplastic stomach tissue from a gastric adenocarcinoma patient are mostly BG4-negative, with haematoxylin counterstaining (blue) readily apparent. Scale bar corresponds to 50 µm. **B**. Adenocarcinoma tissue from the same patient in **A** shows extensive numbers of BG4-positive nuclei (brown). **C**. The nuclei of non-neoplastic stomach tissue from a signet ring cell carcinoma patient are mostly BG4-negative. **D**. Signet ring cell carcinoma tissue from the same patient in **C** shows many BG4-positive nuclei. **E**. Quantification of the number of BG4-positive nuclei in the matched stomach cancers and background non-neoplastic tissues from two different patients. Error bars represent the s.e.m. calculated when duplicate TMA cores were available. These results indicate that there are more G-quadruplex structures in the nuclei of stomach cancer tissue than in non-neoplastic tissue from the same individual.

**Figure 5:**
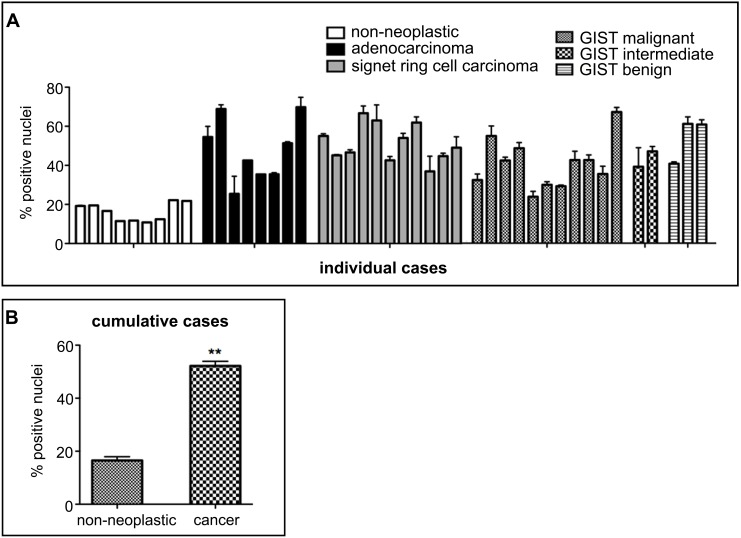
Increased incidence of G-quadruplex-positive cell nuclei across human stomach cancers. Confirmation of the increased presence of BG4-positive nuclei in a range of stomach cancer tissues compared to background non-neoplastic tissues including unmatched cases. **A**. The nuclei of non-neoplastic stomach tissues are largely BG4-negative. Cell nuclei were counterstained with haematoxylin (blue). Scale bar corresponds to 50 µm. **B**. Gastric adenocarcinoma tissue shows the extensive presence of BG4-positive nuclei (brown). **C**. Signet ring cell carcinoma shows many BG4-positive nuclei. **D**. Gastrointestinal stromal tumors (GIST) are largely BG4-positive (for the survey of stomach cancer only unmatched GIST tissues were available). **E**. Quantification of the number of BG4-positive nuclei in individual stomach tissues including unmatched TMA cores. Non-neoplastic stomach tissue shows less BG4-positive nuclei compared to adenocarcinoma, signet ring carcinoma or GIST. Each column corresponds to a single tissue sample and error bars represent the s.e.m. calculated when duplicate tissue samples were available. **F**. Overall quantification of the number of BG4-positive nuclei in all non-neoplastic and stomach liver tissues. Error bars represent the s.e.m. **P<0.001, n = 10 and 34 for non-neoplastic and cancer cores, respectively. These results confirm the generality of more extensive G-quadruplex formation in the stomach cancers compared t­o non-neoplastic tissues.

Similar to the increase of G-quadruplex incidence in liver cancer tissue, a greater than 3-fold increase in the number of BG4-positive nuclei was observed in stomach adenocarcinoma and signet ring cell carcinoma compared with background non-neoplastic tissue taken from the same individual (Figure 4). A wider-ranging analysis of stomach cancer and non-neoplastic tissues including unmatched cases reaffirmed the increase in the number of BG4-positive nuclei seen in stomach cancer compared with non-neoplastic tissue (Figure 5). Indeed, in individual stomach cancer sub-types, adenocarcinomas (originating from glandular epithelia), signet ring cell carcinomas (adenocarcinomas characterized by mucin deposition) and gastrointestinal stromal tumors (GIST, KIT-expressing sarcomas of mesenchymal origin), all showed greater number of BG4-positive nuclei compared to non-neoplastic stomach tissue (Figure 5A). When all cases were considered together, ∼3.1-fold more BG4-positive nuclei were found in cancer versus non-neoplastic tissues (Figure 5B, P<0.001). It is notable that several stomach tumor sub-types, which have different etiologies and progression, are all characterized by increase in BG4 staining. These results are therefore highly suggestive of a general link between an increased presence of G-quadruplex structures and stomach cancer development.

In the liver and stomach TMAs, some cancer tissue cores showed little or no quantifiable BG4 staining, perhaps reflecting an issue in epitope stability during surgical biopsy collection or alternatively pointing to a real variability in G-quadruplex formation in these particular tissues. It is nevertheless of note that in no case was any non-neoplastic liver or stomach tissue extensively BG4-positive. This latter observation further confirms that the increase in the number of BG4-positive cells seen in the cancer TMA cores reflects a true difference in G-quadruplex presence between the non-neoplastic and malignant states. Analysis of G-quadruplex staining in other cancers may therefore be possible and our preliminary analysis of pancreas tissue suggests that, while BG4 staining in non-neoplastic pancreas is widespread, there is a ∼1.6-fold increase of BG4 nuclear staining in adenocarcinoma tissues (P<0.01, Figure S3).

Overall, our results demonstrating that G-quadruplexes can be visualized by IHC in non-neoplastic and cancer human tissues extend our earlier findings that revealed the presence of G-quadruplex structures in the nuclei of culture cell lines [9]. It is noteworthy that we observed an increase in the number of G-quadruplex-positive cells for some cancer tissues compared to non-neoplastic cases. The data that we present do not in themselves explain why more G-quadruplexes are apparent in the cases of stomach and liver cancers. However, there are several lines of evidence in the literature that suggest a potential association or causative link between G-quadruplexes and mechanisms contributing to cancer development or progression. For example, G-quadruplex structures, if not resolved during DNA replication, may represent fragile sites that promote genome instability, which is a well-known hallmark of cancer [24]. Indeed, G-quadruplex sequence motifs are found at DNA breakpoints that lead to translocations within the BCL2 gene in lymphomas and within the HOX11 gene in T-cell leukemias [25], [26]. Computational analyses have also suggested possible associations of G-quadruplexes and breakpoint regions in various cancers [19].

We hypothesize that an increase in genomic G-quadruplexes in cancer may arise from mutations in enzymes that process G-quadruplexes and/or genomic instability at G-quadruplex sites. For example, Fanconi anaemia, Bloom’s and Werner’s syndromes, all display genome instability with a predisposition to cancer [27]–[29] and result from mutations in DNA helicase enzymes that have G-quadruplex-resolving activity [30]–[32]. Recent genome-wide studies have also highlighted the recognition of G-quadruplex sequences by additional resolving helicases such as ATRX, PIF1 and XPB/D [20]–[22]. Furthermore, PIF1 has been suggested to suppress genomic instability at G-quadruplexes [22], while ATRX loss is associated with chromosomal instability in pancreatic neuroendocrine tumors [33], and XPD mutations disrupt nucleotide excision DNA repair that leads to cancer-prone diseases such as Xeroderma pigmentosum [34].

It appears that liver and stomach cancers are highly heterogeneous and are not characterized by a common genetic signature or a highly represented driving mutation that may simply explain the observed increase in G-quadruplex structures. While many liver cancers are associated with hepatitis B or C infection, the pathological information obtained with the TMAs indicates that there is no correlation with BG4 staining. As part of future work, it will be of value to analyze non-neoplastic and malignant tumor samples for cases where whole-genome sequencing has been employed to elucidate the genetic background of the tumor/non-neoplastic pairs, in order to establish the relationship between genotype and G-quadruplex formation in cancer.

In conclusion, we report the use of the G-quadruplex-specific antibody, BG4, in IHC staining experiments of human non-neoplastic and cancer tissues. This study has enabled us to identify a significantly higher presence of DNA G-quadruplex structures in the nuclei of stomach and liver cancer cells compared to the corresponding non-neoplastic tissues. We hypothesize that these differences might be dependent on alterations of cellular processes that regulate genome stability or changes in the chromatin state at G-quadruplex sites in cancer tissues. Our results support the possibility that cancer cells may provide a window of selectivity that would make them more sensitive than non-neoplastic cells towards a small molecule G-quadruplex-targeting strategy.

## Materials and Methods

Fixation, embedding, sectioning and staining of cell pellets were performed by the histopathology core service at the Cancer Research UK Cambridge Institute. Immunohistochemistry (IHC) was performed using standard methods on an automated Leica Bond platform with minor variations. Briefly, tissues were fixed for 24 h at room temperature (RT) in 10% neutral buffered formalin and processed using a Leica ASP300 tissue processor with de-hydration through a graded ethanol series, clearing in xylene and infiltration with molten paraffin wax. Embedding was performed on a Leica EG1160 Embedding Station and sections were cut at 3 µm with a microtome, floated on a water bath set at ∼45–50°C until smooth and flat, before collection on a microscope slide and drying at 60°C for 1 h. De-waxing and re-hydration were performed using an automated Leica ST5020 multistainer. MDA-MB-231 human breast adenocarcinoma cells were obtained from ATCC LGC Standards. For MDA-MB-231 cell pellets, epitope retrieval was performed at 100°C for 20 min with Bond epitope retrieval Solution 1 (citrate-based buffer pH 6.0) or Bond epitope retrieval solution 2 (Tris/EDTA-based buffer pH 9.0) or at 37°C for 10 min with Bond enzyme pre-treatment kit (100 µg/ml proteinase K in Tris-buffered saline, surfactant and 0.35% ProClin 950) to find the best condition. Commercially available human tissue microarrays with appropriate ethical approval in the country of origin were purchased from Insight Bio UK (for US Biomax Inc. arrays) and BioCat, Germany or Stretton Scientific, UK (for Accumax, Isu Abxis arrays). Epitope retrieval was performed at 100°C for 20 min with citrate buffer. BG4 staining was performed at RT on an automated Leica Bond instrument with a rabbit polymer kit (Leica) following a 15 min incubation with BG4 and a 8 min incubation with an anti-FLAG rabbit polyclonal antibody (Cell Signaling Technology). Slides were then counterstained for 5 min with 0.02% haematoxylin to visualize the cell nuclei. De-hydration and clearing were done on an automated Leica ST5020 multistainer, and mounting was performed on an automated glass coverslipper Leica CV5030. Slides were scanned with an Aperio XT120 slide scanning system (Leica) and images analyzed using the Aperio Imagescope Nuclear v9 software. Statistical analyses and P values were calculated using the Student’s t-test. Frequency-distribution graphs were plotted using GraphPad Prism (GraphPad Software).

## Supporting Information

Figure S1 **BG4 staining on paraffin-embedded MDA-MB-231 cell pellets.** A. Human MDA-MB-231 breast cancer cell pellets were fixed, paraffin-embedded and processed for IHC using the G-quadruplex-specific antibody BG4. Strong BG4 staining (brown) is apparent in cell nuclei following epitope retrieval with Tris/EDTA-based buffer pH 9.0. Scale bar corresponds to 20** µ**m. Nuclei are counterstained with haematoxylin (blue). B. Strong BG4 staining (brown) is apparent in cell nuclei following epitope retrieval with proteinase K. C. No nuclear staining is observed in the absence of the BG4 antibody after Tris/EDTA epitope retrieval. D. No nuclear staining is seen following DNase treatment prior to BG4 staining after epitope retrieval with EDTA. E. High levels of non-specific staining in the absence of BG4 after epitope retrieval with proteinase K.
(TIF)

Figure S2 **Non-neoplastic human tissues show variable BG4 staining.** Non-neoplastic tissues were stained by IHC using the G-quadruplex-specific antibody BG4. **A**. BG4 staining (brown) in the kidney cortex shows a range of nuclear staining intensities in glomeruli and associated structures. Cell nuclei were counterstained with haematoxylin (blue). Scale bar corresponds to 50 µm **B**. Weakly positive BG4 staining is seen in the collecting tubule nuclei of the kidney medulla. **C**. Skin shows a range of BG4 intensities in the epidermis with positive and negative nuclei scattered throughout, whereas the dermis is mostly positive. **D**. Most nuclei in colon are BG4-positive. **E**. In the uterine body, the stratified squamous epithelium is largely BG4-negative whereas positive staining is seen more superficially. **F**. Throughout the breast ductal lobules, both myoepithelial and luminal cells show general strong BG4 staining with only occasional negative cells.
(TIF)

Figure S3 **Elevated presence of G-quadruplex structures in human pancreas cancer tissues.** Non-neoplastic and cancer pancreatic tissues were stained by IHC using the G-quadruplex-specific antibody BG4, and the number of BG4-positive nuclei was scored using Aperio Imagescope software. **A**. The nuclei of non-neoplastic pancreas tissue show moderate BG4 staining (brown) with many unstained nuclei also present. Cell nuclei were counterstained with haematoxylin (blue). Scale bar corresponds to 50 µm. **B**. BG4 staining in pancreatic adenocarcinoma tissue is more extensive with greater intensity. **C**. Overall quantification of the number of BG4-positive nuclei across all non-neoplastic and pancreatic cancer tissues. Error bars represent the s.e.m. *P<0.01, n = 6 and 12 for non-neoplastic and cancer cores, respectively.
(TIF)
