# Supplementary figures and images for: Elevated Levels of G-Quadruplex Formation in Human Stomach and Liver Cancer Tissues
Source: PLoS One. 2014 Jul 17;9(7):e102711. doi: 10.1371/journal.pone.0102711 (PMC4102534; doi:10.1371/journal.pone.0102711)

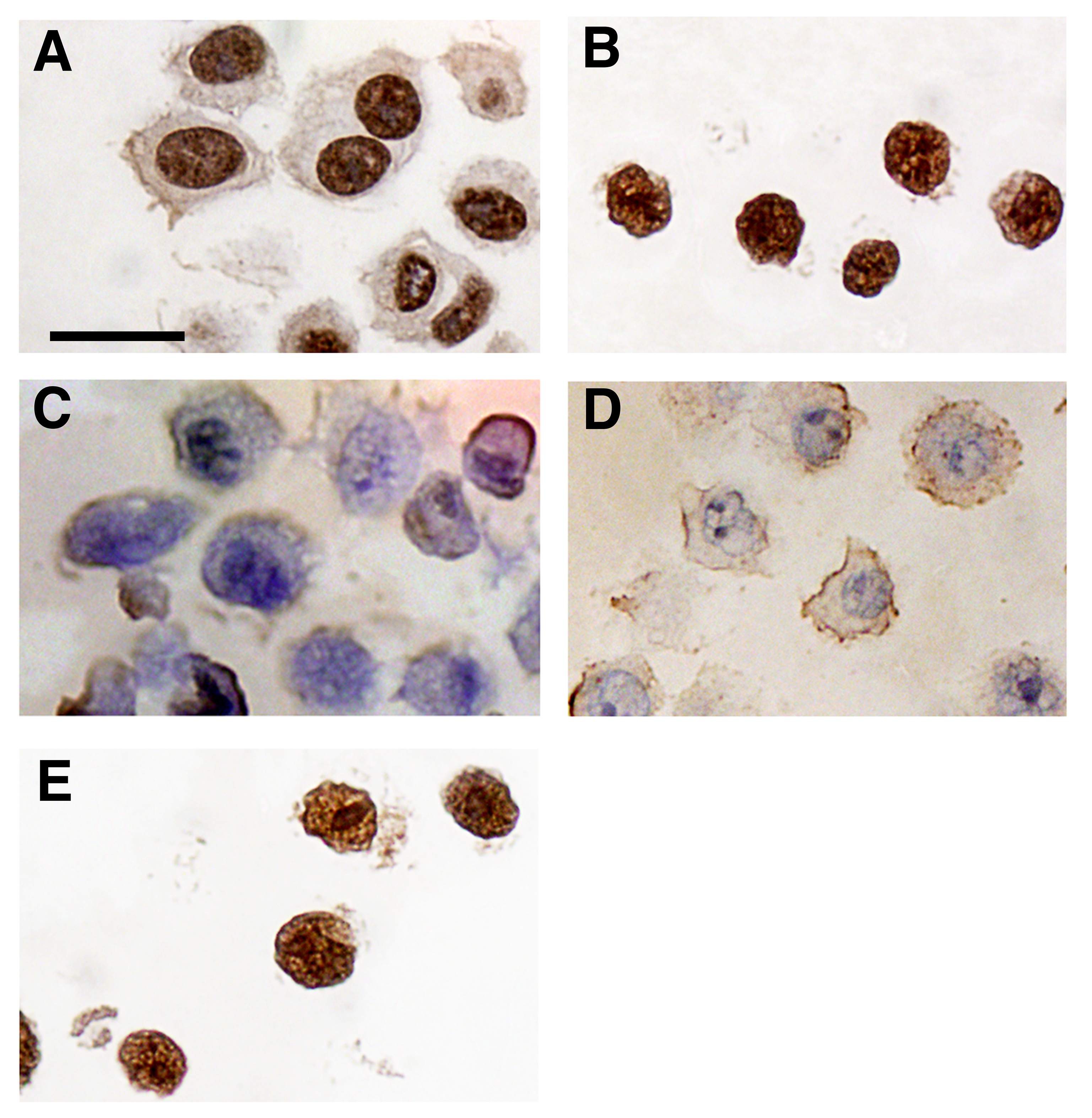

Supplement: Figure S1 — BG4 staining on paraffin-embedded MDA-MB-231 cell pellets. A. Human MDA-MB-231 breast cancer cell pellets were fixed, paraffin-embedded and processed for IHC using the G-quadruplex-specific antibody BG4. Strong BG4 staining (brown) is apparent in cell nuclei following epitope retrieval with Tris/EDTA-based buffer pH 9.0. Scale bar corresponds to 20 µm. Nuclei are counterstained with haematoxylin (blue). B. Strong BG4 staining (brown) is apparent in cell nuclei following epitope retrieval with proteinase K. C. No nuclear staining is observed in the absence of the BG4 antibody after Tris/EDTA epitope retrieval. D. No nuclear staining is seen following DNase treatment prior to BG4 staining after epitope retrieval with EDTA. E. High levels of non-specific staining in the absence of BG4 after epitope retrieval with proteinase K. (TIF) [file pone.0102711.s001.tif]

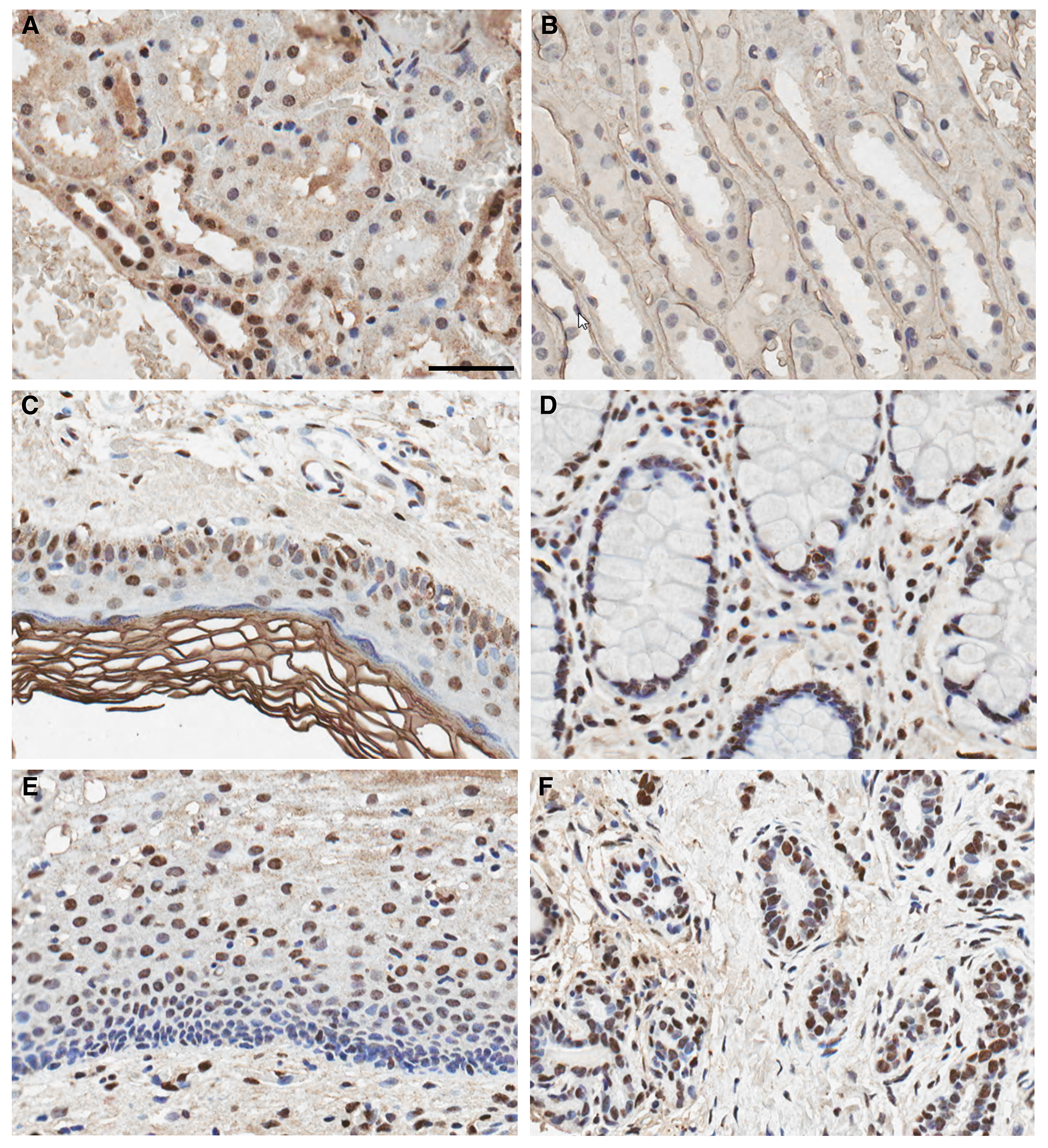

Supplement: Figure S2 — Non-neoplastic human tissues show variable BG4 staining. Non-neoplastic tissues were stained by IHC using the G-quadruplex-specific antibody BG4. A. BG4 staining (brown) in the kidney cortex shows a range of nuclear staining intensities in glomeruli and associated structures. Cell nuclei were counterstained with haematoxylin (blue). Scale bar corresponds to 50 µm B. Weakly positive BG4 staining is seen in the collecting tubule nuclei of the kidney medulla. C. Skin shows a range of BG4 intensities in the epidermis with positive and negative nuclei scattered throughout, whereas the dermis is mostly positive. D. Most nuclei in colon are BG4-positive. E. In the uterine body, the stratified squamous epithelium is largely BG4-negative whereas positive staining is seen more superficially. F. Throughout the breast ductal lobules, both myoepithelial and luminal cells show general strong BG4 staining with only occasional negative cells. (TIF) [file pone.0102711.s002.tif]

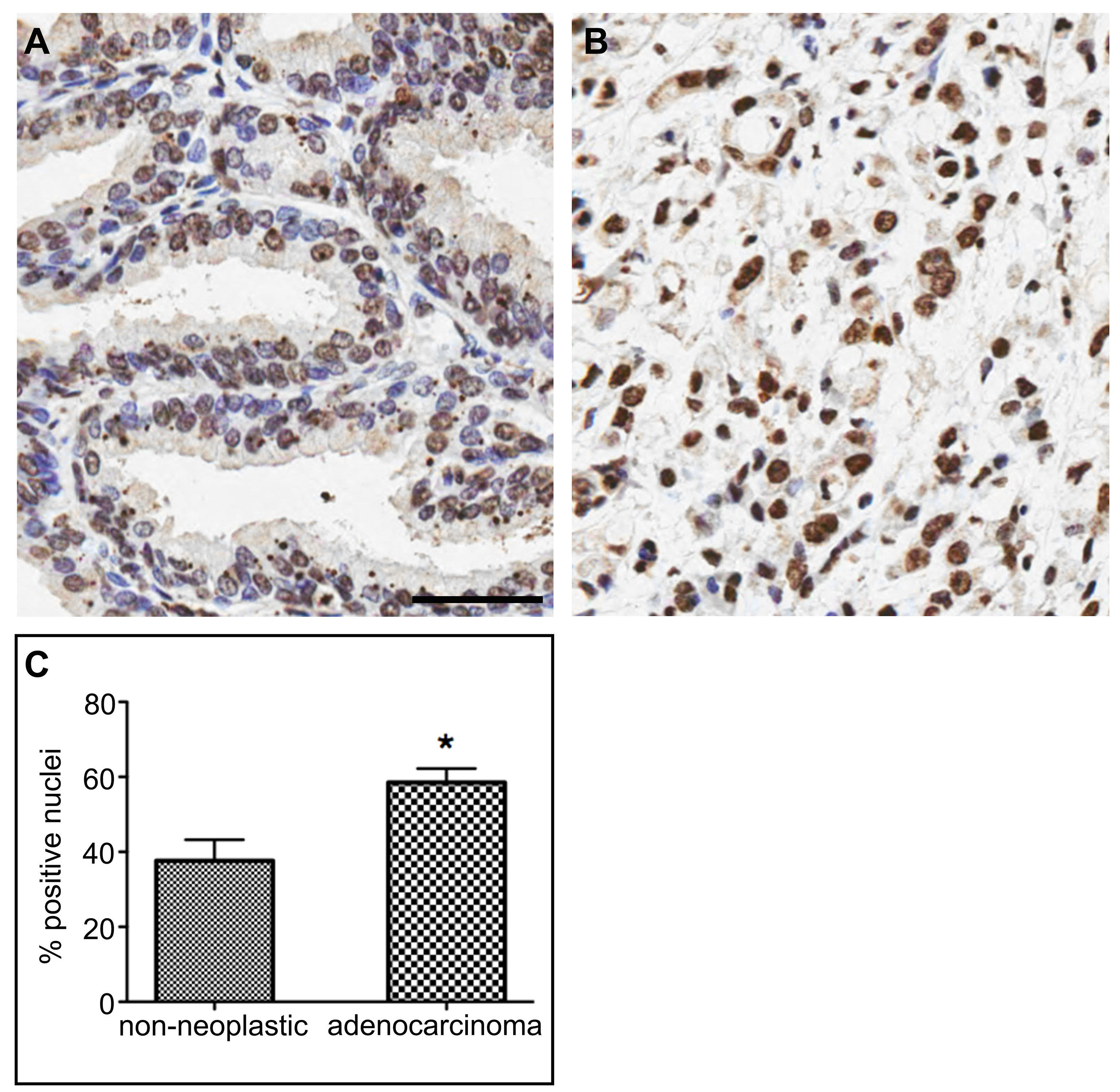

Supplement: Figure S3 — Elevated presence of G-quadruplex structures in human pancreas cancer tissues. Non-neoplastic and cancer pancreatic tissues were stained by IHC using the G-quadruplex-specific antibody BG4, and the number of BG4-positive nuclei was scored using Aperio Imagescope software. A. The nuclei of non-neoplastic pancreas tissue show moderate BG4 staining (brown) with many unstained nuclei also present. Cell nuclei were counterstained with haematoxylin (blue). Scale bar corresponds to 50 µm. B. BG4 staining in pancreatic adenocarcinoma tissue is more extensive with greater intensity. C. Overall quantification of the number of BG4-positive nuclei across all non-neoplastic and pancreatic cancer tissues. Error bars represent the s.e.m. *P<0.01, n = 6 and 12 for non-neoplastic and cancer cores, respectively. (TIF) [file pone.0102711.s003.tif]
